# Beyond Molecular Characterization: The Impact of Age-Adjusted Charlson Comorbidity Index in Glioblastoma Patients Treated with Radio or Radio-Chemotherapy

**DOI:** 10.3390/jcm14217515

**Published:** 2025-10-23

**Authors:** Tamara Ius, Nicola Montemurro, Giuseppe Lombardi, Alberto D’Amico, Luisa Bellu, Alessandro Parisi, Francesco Martino, Giulia Lezzi, Giulia Gobitti, Giulia Gulino, Riccardo Morganti, Giuseppe Catapano, Francesco Acerbi, Luca Denaro, Francesco Pasqualetti, Marco Krengli

**Affiliations:** 1 Academic Neurosurgery, Department of Neurosciences, University of Padova, 35128 Padova, Italy; 2 Department of Neurosurgery, Azienda Ospedaliero Universitaria Pisana (AOUP), 56100 Pisa, Italy; 3 Medical Oncology 1, Veneto Institute of Oncology-IRCCS, 35128 Padua, Italy; 4 UOC Radiotherapy, Istituto Oncologico Veneto, 35128 Padova, Italy; 5 Section of Statistics, Azienda Ospedaliero Universitaria Pisana (AOUP), 56126 Pisa, Italy; 6 Neurosurgical Department, Ospedale del Mare, 80147 Napoli, Italy; 7 Dipartimento di Scienze Chirurgiche, Oncologiche e Gastroenterologiche (DiSCOG), Università di Padova, 35128 Padova, Italy

**Keywords:** glioblastoma, biomarkers, Charlson comorbidity index, geriatric oncology, translational research

## Abstract

**Background**: Glioblastoma (GBM) prognosis has been reported to be influenced by age and comorbidity in several investigations. Identifying factors that contribute to poor survival is crucial to optimizing and personalizing therapeutic strategies. In the present retrospective analysis, we investigated the impact of GBM patient stratification using the age adjusted Charlson Comorbidity Index (ACCI). **Methods**: A total of 165 patients diagnosed with IDH wild-type GBM, treated with post-operative radio or radio-chemotherapy, were evaluated. To assess the impact of comorbidities, patients were stratified into two groups according to their ACCI scores: Group A (ACCI 0–2) and Group B (ACCI >2). The Cox proportional hazards model test was used to compare overall survival (OS) between the two groups of patients and determine whether the presence of comorbidities significantly affected outcomes. Primary and secondary endpoints were OS and progression free survival (PFS), respectively. **Results**: The median follow-up period was 36 months, and the median OS was 14 months (95% CI 12.4–15.5). The univariate analysis evidenced that patients in Group A had a significantly longer OS compared to those in Group B, with median OS times of 18 months (95% CI 16–20) and 12 months (95% CI 10.5–13.5), respectively (*p* = 0.015). The OS remained statistically significant in the multivariate analysis (*p* = 0.015). **Conclusions**: The results of this study indicate that ACCI may serve as an independent prognostic factor in patients with newly diagnosed GBM.

## 1. Introduction

Glioblastoma (GBM) is the most common and aggressive primary malignant brain tumor in adults, characterized by high invasiveness, and poor prognosis despite treatments efforts [1,2]. Over decades of surgical advancements and molecular discoveries, favorable prognosis has continued to be elusive. As a matter of fact, the median survival time has been reported to be less than 15 months, and survival longer than 5 years has been reported for approximately 0.5% of GBM patients [3]. In order to optimize clinical decision making, prognostic variables assessment, including extent of resection (EOR), performance status, O6-methylguanine-DNA methyltransferase (MGMT) promoter methylation status, and age at diagnosis are well-established and extensively utilized [2,3]. Among those, age at diagnosis is a recognized unfavorable prognostic factor in GBM, significantly influencing treatment choices and overall survival rates [4,5,6,7,8,9,10]. Nearly 50% of all GBM cases arise in elderly adults, and this percentage is anticipated to increase with the aging demographic. Furthermore, the prognosis of GBMs is significantly worse in elderly patients, with survival rates ranging from 4 to 9 months. In this clinical context, it is also important to consider that elderly patients often exhibit greater biological frailty and a higher burden of comorbidities, which makes their management particularly challenging [11]. Emerging evidence from small, monocentric, retrospective studies suggests that comorbidities combined with the diagnosis of GBM—alongside age—may contribute to patient outcomes [12,13,14]. However, the precise relationship between overall comorbidity burden and overall survival (OS) remains unclear and has yet to be definitively defined [11,12,14].

In 1987, with the attempt to investigate how multiple concomitant medical disorders impact on survival, researchers lead by Dr. Charlson developed the so called Charlson Comorbidity Index (CCI) [15]. CCI is a clinical tool used to predict mortality by categorizing and scoring a patient’s comorbid conditions. Each condition is assigned a weighted score based on its association with mortality risk. In order to combine the presence of comorbidity with the age, in 1994 the age-adjusted CCI (ACCI) [16] was developed. ACCI builds upon the CCI by adding one point for every decade of life starting at age 51, thereby enhancing its predictive power—particularly in elderly patients—by estimating long-term mortality and clinical outcomes more accurately in those with multiple comorbidities. The new score added points based on the patient’s age, enhancing the predictive accuracy for older populations. This adjustment improved risk stratification, especially in studies involving elderly or multimorbid patients. Using age-adjusted CCI allows for more accurate prognosis and resource planning in both clinical and research settings. ACCI has been widely utilized in epidemiological research, hospital resource planning, and risk stratification for patients with chronic diseases [17,18,19]. In the clinical context of GBM, ACCI could serve as a valuable tool for prognostic evaluation and patient stratification.

In light of the clinical need for novel indices able to integrate the combination of age and comorbidity in patients diagnosed with GBM, the aim of this study is to assess the prognostic value of ACCI in patients with GBM.

## 2. Materials and Methods

### 2.1. Patients

A database of 165 adult patients who underwent surgery for newly diagnosed glioblastoma between January 2015 and December 2020 was analyzed. The case–cohort was defined according to the following inclusion criteria: Age ≥ 18 years; no prior surgical interventions; absence of preoperative chemotherapy or radiotherapy; objective assessment of preoperative tumor volume via MRI in DICOM format, utilizing post-contrast T1-weighted and T2-weighted sequences; objective evaluation of the extent of resection (EOR) on post-contrast T1-weighted MRI sequences; pathological diagnosis in accordance with the 2021 World Health Organization (WHO) Classification of Tumors of the Central Nervous System; assessment of MGMT promoter methylation and IDH1/IDH2 mutation status [20,21]. Cases were omitted from the case–cohort if any of the following criteria were met: surgical approach limited to a simple diagnostic biopsy, incomplete imaging data, inadequate follow-up interval, or the presence of multicentric tumors. Clinical, histological, and molecular data were obtained from medical records at the time of diagnosis.

All patients were treated with postoperative radiotherapy (60 Gy in 30 fractions), with or without temozolomide (75 mg/m^2^/die in combination with radiotherapy and 150/200 mg/m^2^/day as maintenance regimen) [3]. Patients were excluded if they had already diagnosed high-grade gliomas with IDH mutations, a history of prior oncological treatments before surgery, or a previous cancer diagnosis [2,20]. To ensure our patient sample was representative of the broader GBM population and to make multivariate analysis, we evaluated the prognostic impact of MGMT promoter methylation status and the Extent of surgery (EOR).

Progression-free survival (PFS) was assessed using RANO criteria and calculated from the date of diagnosis to the date of disease progression [22]. Overall survival (OS) was defined from the date of surgery to the date of death or last follow-up. The EOR, based on postoperative MRI, was used to categorize patients into two groups. Group 1: total resection (100% of the enhancing tumor on T1-weighted contrast MRI), and Group 2: all others. Similarly, patients were stratified by MGMT promoter methylation status into methylated and unmethylated groups (methylation of the MGMT promoter was assessed on the tumor specimen using the pyrosequencing technique. GBMs were defined as methylated when the average percentage of methylation of CpG islands was ≥8%).

The ACCI was calculated by first determining the CCI, which assigns weighted scores (1 to 6) to 19 medical conditions based on their severity and associated mortality risk. Age adjustments were applied by adding one point for each decade over 50 years (e.g., 50–59 = 1 point; 60–69 = 2 points). The final ACCI score was obtained by summing the CCI and age-related points, providing a comprehensive measure of patient comorbidity and prognosis. Comorbidity data and Karnofsky Performance Status (KPS) were extracted from preoperative medical records. In order to fulfill the main aim of the study and separate our cohort into two different groups based on the presence or not of comorbidity, patients were divided into two groups based on ACCI score. Group A: ACCI score 0–2 (low comorbidity burden), and Group B: ACCI score >2 (high comorbidity burden). The study was approved by local Ethics Committee (Protocol No. 560/2015), and all patients provided written informed consent for surgery.

### 2.2. Statistical Analysis

Categorical data were described with absolute and relative frequency. Overall survival was the primary endpoint of the present study and PFS the secondary one. Survival curves were calculated with Kaplan–Meier method and differences between curves were analyzed by log-rank test. To evaluate the impact of comorbidities on OS and PFS, patients were divided into two distinct groups based on their ACCI scores: Group A (score 0–2) and Group B (score >2). Furthermore, the influence of MGMT methylation status, extent of surgery and ACCI on the survival was assessed using the univariate Cox regression followed by multivariate analysis. The results were expressed as Hazard Ratios (HRs) with 95% confidence Intervals (Cis) and corresponding *p*-values. Significance was set at 0.05 and all analyzes were performed with SPSS v.29 statistics. (https://www.ibm.com/products/spss-statistics, accessed on 22 March 2025).

## 3. Results

Data analysis was conducted in February 2025. A total of 165 consecutive patients with IDH-wildtype GBM, classified according to the 2021 WHO criteria, were included in the study. The median age was 62 years (range: 39–84). All patients met all inclusion criteria. Demographic, clinical, and radiological features of the study population are summarized in Table 1.

The median follow-up period was 36 months (range: 26–63 months), and the median OS was 14 months (95% CI: 12.4–15.5). Eighty-five patients had a KPS equal to or greater than 90 (51.5%), while 80 patients had a KPS equal to or less than 80 (48.5%). MGMT methylation was assessed in all patients, 65 (39.4%) had MGMT promoter not methylated, while in the other 100 (60.6%) it was methylated. A radical removal of the tumor was achieved in 68 patients (41.2%), a partial one (90–99%) in 76 patients (46.1%), and lesser than 90% of the tumor was removed in the other 21 patients (12.7%).

**Figure 1:**
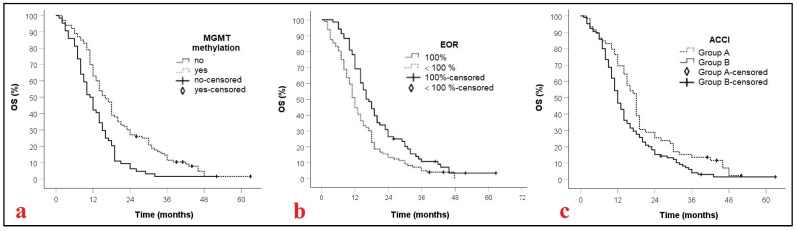
Kaplan–Meier curves displaying OS of GBM patients according to MGMT methylation, *p* = 0.001, (**a**); EOR, *p* = 0.001 (**b**), and ACCI, *p* = 0.015, (**c**). MGMT: methylguanine-DNA methyltransferase; EOR: extent of resection; ACCI: Age adjusted comorbidity index; OS: Overall survival.

Sixty patients (36.4%) had an ACCI score of 0–2, while 105 (63.6%) were graded greater than 2 (Table 2). As shown in Figure 1, univariate analysis revealed that patients in Group A had significantly longer survival than those in Group B, with median OS of 18 months (95% CI: 16–20) and 12 months (95% CI: 10.5–13.5), respectively (*p* = 0.015). This survival difference remained statistically significant in the multivariate analysis.

Additionally, MGMT promoter methylation and radical tumor resection were independently associated with improved OS in the multivariate model. Specifically, MGMT methylation was associated with a hazard ratio (HR) of 0.6 (95% CI: 0.4–0.7, *p* = 0.001), and radical resection with an HR of 1.6 (95% CI: 1.2–2.0, *p* = 0.001) (Table 2).

Progression-free survival (PFS) was also favored patients in Group A based on ACCI, with a median PFS of 11 months (95% CI: 9.5–12.5), compared to 8 months (95% CI: 6.9–9.1) in Group B (*p* = 0.017). This PFS advantage remained statistically significant in multivariate 0.022). Table 3 and Table 4 report frequency associated with the ACCI values and conditions included in the Charlson Comorbidity Index and their corresponding weights, respectively.

## 4. Discussion

In this retrospective study carried out on 165 patients with newly diagnosed GBM, OS and PFS were analyzed based on the stratification of clinical, radiological, and molecular variables. The Key findings indicate that both OS and PFS were significantly influenced by the Age-Adjusted Charlson Comorbidity Index.

Patients were divided into two groups based on their ACCI scores: Group B (scores > 2), which indicated an increased burden of comorbidity, and Group A (scores 0–2), where age was the primary contributing factor. This classification showed significant survival differences, which were validated in multivariate analysis despite the study’s small sample size. Univariate analysis showed that patients in Group A had significantly longer survival respect to those in Group B, with median OS of 18 months (95% CI: 16–20) and 12 months (95% CI: 10.5–13.5), respectively (*p* = 0.015). These differences persisted even after adjusting for key-factors such as EOR and the MGMT status. The response to radio-chemotherapy was also longer in patients with a low score of ACCI, patients in Group A showed a median PFS of 11 months (95% CI: 9.5–12.5), compared to 8 months (95% CI: 6.9–9.1) in Group B (*p* = 0.017). This PFS advantage remained statistically significant in multivariate analysis (HR: 1.366; 95% CI: 1.045–1.784; *p* = 0.022). In addition, this study confirms that the EOR and MGMT promoter methylation status are treatment-independent prognostic factors, supporting the central role of surgical debulking and chemoradiotherapy in the initial management of GBM. Overall, our findings demonstrated that ACCI is a useful tool for patient risk stratification in this clinical scenario. By integrating comorbid illnesses and chronological age, the ACCI offers thus an easy and comprehensive way to assess patient fitness. Even while age is a common stratification characteristic in GBM, it might not fully account for physiological variability or functional reserve, particularly in older patients.

ACCI could improve tailored treatment planning and allow for more sophisticated risk assessment by combining the two dimensions. Although age is often considered an independent prognostic factor in GBM patients, its predictive value may be confounded or hidden by patient fitness. Emerging evidence suggests that fit elderly patients can achieve survival outcomes comparable to those of younger individuals [23]. Furthermore, the interplay between age, comorbidities, and overall patient frailty remains nearly underexplored. For example, Brown et al. [13] retrospectively analyzed 517 GBM patients and found that median OS varied markedly by age group: 5.6 months in patients over 70 (95% CI: 3.9–6.7), 14 months for those aged 50–59 (95% CI: 11.5–16.4), and 16.7 months for those under 50 (95% CI: 14.5–20.7). Similar trends were observed by Chen et al. [23] and Poszat et al. [24]. Beyond age, several studies have explored the prognostic significance of comorbidities. Villani et al. [11] examined comorbidities using CCI in GBM patients and found an association between higher comorbidity burden and worse survival outcomes. However, their cohort was heterogeneous regarding treatment and did not uniformly adhere to the 2021 WHO GBM classification. In a more recent multicenter investigation, Ius et al. [25] emphasized that age alone should not dictate treatment decisions for elderly GBM patients. They advocated a personalized approach, incorporating performance status, comorbidities, and general health to guide therapy, ultimately improving survival and quality of life. In exploring vascular comorbidity, our team also investigated in a small cohort of patients diagnosed with GBM and treated with post-operative radio-chemotherapy the potential of the calcium index measured on internal carotid artery wall as a prognostic biomarker. Univariate and multivariate analyses showed that patients with low calcium scores—indicative of reduced cardiovascular risk—had significantly improved OS (29 months; 95% CI: 16–41) compared to those with high calcium scores (13 months; 95% CI: 10–16), *p* = 0.011 [26].

The prognostic value of ACCI has already been evaluated across various solid tumors. For example, in 2023, Telli et al. [27] studied 151 patients with trunk and extremity sarcomas and found ACCI to be an independent prognostic factor for OS (*p* = 0.001), with higher scores correlating with worse outcomes. Notably, their study defined ACCI scores of 1–3 as low, whereas in the present analysis we used 1–2. Wu et al. [18] evaluated 5643 colorectal cancer patients using data from Taiwan’s National Health Insurance Research Database and confirmed ACCI as an independent predictor of survival.

In the GBM setting, Barz et al. [12] investigated 120 patients with recurrent disease and found no prognostic value of ACCI. The authors identified EOR and KPS as prognostic factors at both univariate and multivariate analyses. Although it represented the main focus of the analysis, ACCI was not shown as a prognostic factor in univariate or multivariate analyses. However, the data from this study should be evaluated considering the selection biases made by the neurosurgeon. For this analysis, not all patients with GBM or GBM recurrence were evaluated, but only those considered suitable for the second surgery at the time of recurrence (thus an inference was impossible; the sample was not representative of all patients diagnosed with GBM). Additionally, only 27 patients had received postoperative radio-chemotherapy according to the Stupp protocol, further limiting the results of that study [28].

Our results are instead consistent with those reported by Schneider et al. [29] and Ening et al. [30], who demonstrated that a high total comorbidity burden (CCI ≥ 3) is an independent negative prognostic factor, while individual conditions fail to retain significance in multivariable models. Further supporting this interpretation, recent studies in geriatric oncology conceptualize frailty as a dynamic and syndromic construct characterized by reduced physiological reserve and multisystem vulnerability rather than the mere presence of one or two diseases [31,32,33,34,35]. This is particularly relevant in the context of GBM, where age-related immune-senescence, sarcopenia, and polypharmacy may interact synergistically with tumor biology and treatment toxicity [36]. In summary, our study contributes to the growing body of evidence suggesting that aggregated comorbidity burden—rather than single disease entities—is more clinically relevant in predicting prognosis among GBM patients.

The multifaceted aspect of patient fragility was highlighted by Schneider et al.’s demonstration in a sample of older GBM patients that a higher total comorbidity burden, rather than individual disorders, corresponded with a lower OS [29]. In a similar vein, Ening et al. [30] found that no single comorbidity stood out as a dominant factor, but that a CCI score of ≥3 was an independent predictor of poor survival in newly diagnosed GBM. This is consistent with the research on geriatric oncology, which increasingly views frailty as a syndromic state caused by a reduction in physiological reserve in several systems rather than being caused by a single pathology [30].

Given our findings and the growing body of literature, prospective translational studies are warranted to evaluate the potential predictive role of ACCI in GBM treatment responses. Moreover, enhancing translational research could help uncover the biological mechanisms underlying poorer prognosis in high-ACCI patients, enabling more targeted therapeutic strategies. Additionally, the prognostic relevance of molecular and immunohistochemical markers in GBM continues to emerge. These data highlight the need to integrate clinical indices such as the ACCI with molecular profiles to refine risk stratification and guide personalized treatment options.

### Limitations of the Study

Despite the promising results, this study holds several limitations. We acknowledge the absence of an external validation cohort as an important limitation related to the retrospective, single-center design of this study. The homogeneity of our cohort—characterized by a consistent diagnosis of IDH-wildtype GBM per WHO 2021 criteria and standardized postoperative treatment—enhances the internal validity of our findings, thereby reducing selection bias. Additionally, the ACCI grouping criteria (1–2 vs. >2) have not been universally adopted and is different from previous research. Cross-study comparability may be impacted by this; thus, future studies should try to find clinically validated thresholds using receiver operating characteristic (ROC) analyses. The potential association between ACCI and treatment-related toxicity represents an important key-point for future research. Unfortunately, due to the retrospective design of this investigation, we were unable to systematically collect toxicity data for radiotherapy and chemotherapy. Prospective studies incorporating detailed toxicity profiles may facilitate the refinement of treatment plans for GBM patients at higher risk.

Future multicenter studies that include external validation cohorts and frailty-specific evaluations will be crucial to validate the generalizability and therapeutic relevance of our findings.

## 5. Conclusions

This study supports the use of the ACCI as an independent prognostic factor to refine prognosis in elderly patients with newly diagnosed GBM. In light of the advanced age and clinical heterogeneity typically observed in this population, future prospective studies with larger sample sizes are needed to validate these results.

## Figures and Tables

**Table 1:** Baseline Patient’s characteristics.

Patient Characteristics	
Gender	Male 108 (65.5%)
	Female 57 (34.5%)
Median age years (range)	62 (39–84)
MGMT meth	Yes 65 (39.4%)
	No 100 (60.6%)
EOR	100%: 68 pts (41.2%)
	90–99%: 76 pts (46.1%)
	<90%: 21 pts (12.7%)
ACCI	1–2: 60 pts (36.4%)
	>2: 105 pts (63.6%)
KPS	90–100: 85 pts (51.5%)
	80: 48 pts (48.5%
	60–70: 32 pts (19.4%)
Chemo	Yes: 158 pts (95.8%)
	No: 7 pts (4.2%)

**Table 2:** Table shows the influence of different factors on the OS and PFS rates by multivariate analysis. MGMT: methylguanine-DNA methyltransferase; ACCI: Age-Adjusted Charlson Comorbidity Index; EOR: Extent of resection.

Multivariate Analysis of OS Predictive Factors					
Factor	RC	HR	95% CI-Lower	95% CI-Upper	*p*-Value
MGMT: Meth, no meth	−0.581	0.559	0.399	0.783	0.001
EOR: 100%, <100%	0.452	1.572	1.213	2.037	0.001
ACCI: 0–2, >2	0.416	1.516	1.084	2.121	0.015
**Multivariate Analysis of PFS Predictive Factors**					
**Factor**	**RC**	**HR**	**95% CI-Lower**	**95% CI** **-Upper**	***p*-Value**
MGMT: Meth, no meth	−0.638	0.529	0.371	0.752	<0.001
EOR: 100%, <100%	0.346	1.414	1.009	1.981	0.044
ACCI: 0–2, >2	0.312	1.366	1.045	1.784	0.022

**Table 3:** Table of frequency associated with the ACCI values.

ACCI	Frequency	%
0	5	3
1	16	9.7
2	39	23.6
3	29	17.6
4	27	16.4
5	20	12.1
6	17	10.3
7	4	2.4
8	3	1.8
9	3	1.8
10	2	1.2

**Table 4:** Conditions included in the Charlson Comorbidity Index and their corresponding weights.

Condition	Weight
Myocardial infarction	1
Congestive heart failure	1
Peripheral vascular disease	1
Cerebrovascular disease (e.g., stroke, TIA)	1
Dementia	1
Chronic pulmonary disease (e.g., COPD)	1
Connective tissue disease (e.g., RA, SLE)	1
Peptic ulcer disease	1
Mild liver disease	1
Diabetes without end-organ damage	1
Diabetes with end-organ damage	2
Hemiplegia or paraplegia	2
Moderate or severe renal disease	2
Any malignancy (including leukemia and lymphoma)	2
Moderate or severe liver disease	3
Metastatic solid tumor	6
AIDS	6

## Data Availability

The original contributions presented in this study are included in the article. Further inquiries can be directed to the corresponding authors.

## Abbreviations

The following abbreviations are used in this manuscript:

MGMT	methylguanine-DNA methyl-transferase
meth	methylated
ACCI	methylguanine-DNA methyltransferase
KPS	Karnofsky performance score
CT	chemotherapy
PTS	patients
CCI	Charlson Comorbidity Index
ACCI	age adjusted Charlson Comorbidity Index

## References

[1] Grochans S., Cybulska A.M., Simińska D., Korbecki J., Kojder K., Chlubek D., Baranowska-Bosiacka I. (2022). Epidemiology of Glioblastoma Multiforme-Literature Review. Cancers.

[2] Weller M., van den Bent M., Preusser M., Le Rhun E., Tonn J.C., Minniti G., Bendszus M., Balana C., Chinot O., Dirven L. (2021). EANO guidelines on the diagnosis and treatment of diffuse gliomas of adulthood. Nat. Rev. Clin. Oncol..

[3] Stupp R., Mason W.P., van den Bent M.J., Weller M., Fisher B., Taphoorn M.J.B., Belanger K., Brandes A.A., Marosi C., Bogdahn U. (2005). Radiotherapy plus concomitant and adjuvant temozolomide for glioblastoma. N. Engl. J. Med..

[4] Flanigan P.M., Jahangiri A., Kuang R., Truong A., Choi S., Chou A., Molinaro A.M., McDermott M.W., Berger M.S., Aghi M.K. (2018). Developing an Algorithm for Optimizing Care of Elderly Patients with Glioblastoma. Neurosurgery.

[5] Chaichana K.L., Chaichana K.K., Olivi A., Weingart J.D., Bennett R., Brem H., Quiñones-Hinojosa A. (2011). Surgical outcomes for older patients with glioblastoma multiforme: Preoperative factors associated with decreased survival. Clinical article. J. Neurosurg..

[6] Babu R., Komisarow J.M., Agarwal V.J., Rahimpour S., Iyer A., Britt D., Karikari I.O., Grossi P.M., Thomas S., Friedman A.H. (2016). Glioblastoma in the elderly: The effect of aggressive and modern therapies on survival. J. Neurosurg..

[7] Scott J.G., Suh J.H., Elson P., Barnett G.H., Vogelbaum M.A., Peereboom D.M., Stevens G.H.J., Elinzano H., Chao S.T. (2011). Aggressive treatment is appropriate for glioblastoma multiforme patients 70 years old or older: A retrospective review of 206 cases. Neuro Oncol..

[8] Tanaka S., Meyer F.B., Buckner J.C., Uhm J.H., Yan E.S., Parney I.F. (2013). Presentation, management, and outcome of newly diagnosed glioblastoma in elderly patients. J. Neurosurg..

[9] Álvarez de Eulate-Beramendi S., Álvarez-Vega M.A., Balbin M., Sanchez-Pitiot A., Vallina-Alvarez A., Martino-González J. (2016). Prognostic factors and survival study in high-grade glioma in the elderly. Br. J. Neurosurg..

[10] Molinaro A.M., Hervey-Jumper S., Morshed R.A., Young J., Han S.J., Chunduru P., Zhang Y., Phillips J.J., Shai A., Lafontaine M. (2020). Association of Maximal Extent of Resection of Contrast-Enhanced and Non-Contrast-Enhanced Tumor with Survival Within Molecular Subgroups of Patients with Newly Diagnosed Glioblastoma. JAMA Oncol..

[11] Villani V., Tanzilli A., Telera S.M., Terrenato I., Vidiri A., Fabi A., Zucchella C., Carapella C.M., Marucci L., Casini B. (2019). Comorbidities in elderly patients with glioblastoma: A field-practice study. Future Oncol..

[12] Barz M., Bette S., Janssen I., Aftahy A.K., Huber T., Liesche-Starnecker F., Ryang Y.-M., Wiestler B., Combs S.E., Meyer B. (2022). Age-adjusted Charlson comorbidity index in recurrent glioblastoma: A new prognostic factor?. BMC Neurol..

[13] Brown N.F., Ottaviani D., Tazare J., Gregson J., Kitchen N., Brandner S., Fersht N., Mulholland P. (2022). Survival Outcomes and Prognostic Factors in Glioblastoma. Cancers.

[14] Reihanian Z., Abbaspour E., Zaresharifi N., Karimzadhagh S., Mahmoudalinejad M., Sourati A., Farzin M., EslamiKenarsari H. (2024). Impact of Age and Gender on Survival of Glioblastoma Multiforme Patients: A Multicenter Retrospective Study. Cancer Rep..

[15] Charlson M.E., Pompei P., Ales K.L., MacKenzie C.R. (1987). A new method of classifying prognostic comorbidity in longitudinal studies: Development and validation. J. Chronic Dis..

[16] Charlson M., Szatrowski T.P., Peterson J., Gold J. (1994). Validation of a combined comorbidity index. J. Clin. Epidemiol..

[17] Zhang N., Lin Q., Jiang H., Zhu H. (2023). Age-adjusted Charlson Comorbidity Index as effective predictor for in-hospital mortality of patients with cardiac arrest: A retrospective study. BMC Emerg. Med..

[18] Wu C.C., Hsu T.-W., Chang C.-M., Yu C.-H., Lee C.-C. (2015). Age-adjusted Charlson comorbidity index scores as predictor of survival in colorectal cancer patients who underwent surgical resection and chemoradiation. Medicine.

[19] Ius T., Somma T., Altieri R., Angileri F.F., Barbagallo G.M., Cappabianca P., Certo F., Cofano F., D’eLia A., Della Pepa G.M. (2020). Is age an additional factor in the treatment of elderly patients with glioblastoma? A new stratification model: An Italian Multicenter Study. Neurosurg. Focus.

[20] McNamara C., Mankad K., Thust S., Dixon L., Limback-Stanic C., D’aRco F., Jacques T.S., Löbel U. (2022). 2021 WHO classification of tumours of the central nervous system: A review for the neuroradiologist. Neuroradiology.

[21] Pasqualetti F., Malfatti G., Cantarella M., Gonnelli A., Montrone S., Montemurro N., Gadducci G., Giannini N., Pesaresi I., Perrini P. (2022). Role of magnetic resonance imaging following postoperative radiotherapy in clinical decision-making of patients with high-grade glioma. Radiol. Med..

[22] Wen P.Y., Macdonald D.R., Reardon D.A., Cloughesy T.F., Sorensen A.G., Galanis E., DeGroot J., Wick W., Gilbert M.R., Lassman A.B. (2010). Updated response assessment criteria for high-grade gliomas: Response assessment in neuro-oncology working group. J. Clin. Oncol..

[23] Chen J.W., Zhou C.F., Lin Z.X. (2015). The influence of different classification standards of age groups on prognosis in high-grade hemispheric glioma patients. J. Neurol. Sci..

[24] Paszat L., Laperriere N., Groome P., Schulze K., Mackillop W., Holowaty E. (2001). A population-based study of glioblastoma multiforme. Int. J. Radiat. Oncol. Biol. Phys..

[25] Ius T., Pignotti F., Della Pepa G.M., La Rocca G., Somma T., Isola M., Battistella C., Gaudino S., Polano M., Bo M.D. (2020). A Novel Comprehensive Clinical Stratification Model to Refine Prognosis of Glioblastoma Patients Undergoing Surgical Resection. Cancers.

[26] Pasqualetti F., Gabelloni M., Faggioni L., Aquaro G.D., De Vietro F., Mendola V., Spina N., Frey J., Montemurro N., Cantarella M. (2024). Glioblastoma and Internal Carotid Artery Calcium Score: A Possible Novel Prognostic Partnership?. J. Clin. Med..

[27] Telli T.A., Alan O., Demircan N.C., Sariyar N., Arikan R., Basoglu T., Yasar A., Celebi A., Isik S., Sofulu O. (2023). Age-adjusted Charlson Comorbidity Index is a valuable prognostic tool in operable soft tissue sarcoma of trunk and extremities. Orthop. Traumatol. Surg. Res..

[28] Pasqualetti F., Barberis A., Zanotti S., Montemurro N., De Salvo G.L., Soffietti R., Mazzanti C.M., Ius T., Caffo M., Paiar F. (2023). The impact of survivorship bias in glioblastoma research. Crit. Rev. Oncol. Hematol..

[29] Schneider M., Potthoff A.-L., Scharnböck E., Heimann M., Schäfer N., Weller J., Schaub C., Jacobs A.H., Güresir E., Herrlinger U. (2020). Newly diagnosed glioblastoma in geriatric (65 +) patients: Impact of patients frailty, comorbidity burden and obesity on overall survival. J. Neurooncol..

[30] Ening G., Osterheld F., Capper D., Schmieder K., Brenke C. (2015). Charlson comorbidity index: An additional prognostic parameter for preoperative glioblastoma patient stratification. Charlson comorbidity index: An additional prognostic parameter for preoperative glioblastoma patient stratification. J. Cancer Res. Clin. Oncol..

[31] Conrad K., Löber-Handwerker R., Hazaymeh M., Rohde V., Malinova V. (2024). Personalized prognosis stratification of newly diagnosed glioblastoma applying a statistical decision tree model. J. Neurooncol..

[32] Rabin E.E., Huang J., Kim M., Mozny A., Lauing K.L., Penco-Campillo M., Zhai L., Bommi P., Mi X., Power E.A. (2024). Age-stratified comorbid and pharmacologic analysis of patients with glioblastoma. Brain Behav. Immun. Health.

[33] Aly A., Singh P., Korytowsky B., Ling Y.-L., Kale H.P., Dastani H.B., Botteman M.F., Norden A.D. (2020). Survival, costs, and health care resource use by line of therapy in US Medicare patients with newly diagnosed glioblastoma: A retrospective observational study. Neurooncol. Pract..

[34] Wank M., Schilling D., Schmid T.E., Meyer B., Gempt J., Barz M., Schlegel J., Liesche F., Kessel K.A., Wiestler B. (2018). Human Glioma Migration and Infiltration Properties as a Target for Personalized Radiation Medicine. Cancers.

[35] Chandra A., Rick J.W., Ore C.D., Lau D., Nguyen A.T., Carrera D., Bonte A., Molinaro A.M., Theodosopoulos P.V., McDermott M.W. (2018). Disparities in health care determine prognosis in newly diagnosed glioblastoma. Neurosurg. Focus..

[36] Krenzlin H., Jankovic D., Alberter C., Kalasauskas D., Westphalen C., Ringel F., Keric N. (2021). Frailty in Glioblastoma Is Independent from Chronological Age. Front. Neurol..

